# Resolution of grammatical tense into actual time, and its application in Time Perspective study in the tweet space

**DOI:** 10.1371/journal.pone.0211872

**Published:** 2019-02-20

**Authors:** Sabyasachi Kamila, Mohammad Hasanuzzaman, Asif Ekbal, Pushpak Bhattacharyya

**Affiliations:** 1 Department of Computer Science and Engineering, Indian Institute of Technology Patna, Bihar, India; 2 ADAPT Centre, School of Computing, Dublin City University, Dublin, Ireland; Radboud Universiteit, NETHERLANDS

## Abstract

Time Perspective (TP) is an important area of research within the ‘psychological time’ paradigm. TP, or the manner in which individuals conduct themselves as a reflection of their cogitation of the *past*, the *present*, and the *future*, is considered as a basic facet of human functioning. These perceptions of time have an influence on our actions, perceptions, and emotions. Assessment of TP based on human language on Twitter opens up a new avenue for research on *subjective view* of time at a large scale. In order to assess TP of users’ from their tweets, the foremost task is to resolve grammatical tense into the underlying temporal orientation of tweets as for many tweets the tense information, and their temporal orientations are not the same. In this article, we first resolve grammatical tense of users’ tweets to identify their underlying temporal orientation: past, present, or future. We develop a minimally supervised classification framework for temporal orientation task that enables incorporating linguistic knowledge into a deep neural network. The temporal orientation model achieves an accuracy of 78.7% when tested on a manually annotated test set. This method performs better when compared to the state-of-the-art technique. Secondly, we apply the classification model to classify the users’ tweets in either of the *past*, *present* or *future* categories. Tweets classified this way are then grouped for each user which gives rise to unidimensional TP. The valence (*positive*, *negative*, and *neutral*) is added to the temporal orientation dimension to produce the bidimensional TP. We finally investigate the association between the Twitter users’ unidimensional and bidimensional TP and their age, education and six basic emotions in a large-scale empirical manner. Our analysis shows that people tend to think more about the past as well as more positive about the future when they age. We also observe that future-negative people are less joyful, more sad, more disgusted, and more angry while past-negative people have more fear.

## Introduction

In the era of data science, written languages on social media have strengthen the research on social science in an unprecedented manner. This allows the social science research to be more data-driven [1]. The social media content has been influential for analyzing different user attributes from their language use. The studies include age, gender prediction [2, 3], psychological well being [4–12], and a host of other behavioral, psychological and medical phenomena [13–16]. However, a very few studies exist which analyze these factors using the social media users’ cognitive structure like Time Perspective (TP).

Time has been carefully studied in a multitude of ways by philosophers, sociologists, anthropologists, and psychologists. One central point of the age-old philosophical debate is whether the time is subjective or objective. The objective paradigm has seen time as a physical phenomenon- something measurable, continuous, homogeneous and universal [17, 18]. The subjective view of time perceives it as an internal, subjective phenomenon, often called ‘psychological time’, ‘lived time’ or ‘time as it is processed by the human mind’ [19]. Within the subjective notion of time, research has focused on time estimation, subjective duration of experience or time perception, time personality, time congruity, time urgency, time intensity, polychronicity and monochronicity, time structure and perceived time use [20–23].

Human TP can be defined as ‘a cognitive operation that implies both an emotional reaction to time zones (such as future, present or past) and a preference for locating action in some temporal zone’ [24]. The most dominant measure of TP, the Zimbardo Time Perspective Inventory (ZTPI) modified from the Stanford Time Perspective Inventory is argued to have addressed the shortcomings of the previous scales [25]. The ZTPI scale considers TP as bidimensional constructs by taking into account both the different temporal orientations (past, present, and future) and the valence dimension. It consists of five factors: Past-Negative, Past-Positive, Present-Hedonistic, Present-Fatalistic and Future. Although ZTPI has emerged as the leading measure of TP, some theoretical and empirical studies have pointed out the drawbacks to it due to the lack of valence in the future temporal orientation [26–29].

The formation of TP is believed to be heavily influenced by various factors including upbringing, socialization, culture, education, life stressors, and other situational factors that contribute to the formation of TP orientation [25, 30, 31]. It is also claimed to be a stable personality characteristic [25]. The TP is influential as it is a non-conscious process that gives orderliness to the events by associating them to different time frames. These cognitive frames are utilized to encode, accumulate and recollect the past experiences and influence the formation of the thought processes, behaviours, and other human attributes [32].

Social science and psychological studies show that TP orientation has a profound impact on the aspects of our behavior, attitudes, emotion, educational achievements, health, sleep and dreaming patterns, choice of food, romantic partner choices, sexual behavior, risk-taking, academic goal setting, interpersonal relations, organizational behavior and perceived time pressure among the other factors [25, 30, 32–36]. Previous psychological studies also demonstrate the association between TP and different human attributes such as age, gender, education [37–39], psychological traits [1], depression, anxiety, anger, aggression [40] and happiness [41].

### Measuring TP

Traditionally, TP is assessed by the self-report questionnaires, more dominantly by ZTPI [25]. In ZTPI measure, the respondents rate the statements in different sub-scales which are used to measure different time-perspective sub-scales. The sub-scales are Past-Positive (“It gives me pleasure to think about the past.”), Past-Negative (“I think about the bad things that have happened to me in the past.”), Present-Hedonistic (“Taking risks keeps my life from becoming boring.”), Present-Fatalistic (“Often luck pays off better than hard work.”) and Future (“I complete projects on time by making steady progress,”).

Although this self-report measure is easy to administer, it highly overlaps with the self-reported measures of personality traits (e.g., future vs conscientious) [42]. The evaluation strategies like ZTPI’s Past-Negative includes different aspects of rating positive and negative experiences [25]. But maybe people’s unpleasant experiences like sadness, depression causing these relations. Thus these measures have some limitations on the actual contribution of TP to human tendencies. These self-report measures are also a very time-consuming process.

In contrast, language-based assessments can be effectively used to alternate existing TP measures using the insights from natural language processing (NLP). Unlike self-report measures where participants are driven by selective questionnaires, a majority of social media users write true positive information regarding themselves and express their true characteristics [43]. Twitter allows easy access to the vast amount of natural language text for research purpose in comparatively lesser cost. Also, there are many tools freely available for accessing tweets of million users. Twitter data is noisy (informal, ungrammatical constructions, etc.) which makes it one of the most challenging and complex text forms to process. Thus, tweets have easy accessibility, challenges to handle and potential information to study human tendencies.

In recent time, TP has been measured from the language people use in social media and correlated with different user attributes [42, 44, 45]. In [42, 44], temporal orientation classifiers are built with supervised learning technique on the manually annotated data. The authors then correlate the user-level temporal orientation with users’ age, gender, IQ, satisfaction with life, depressive symptoms, and Big-five personality factors (conscientiousness, openness, extraversion, neuroticism, and agreeableness). Another study reported in [45] measures the temporal orientation in the same manner as the previous. In this research the authors built temporal classifier by a keyword-based approach to measure the user-level temporal orientation and correlate it with the users’ income level.

In all these studies, TP has been perceived as a unidimensional construct where only the temporal orientation dimension is considered. On the contrary, we are interested in both the unidimensional and bidimensional TP from the language people use in Twitter. Earlier psychological study shows that *future* oriented people are usually joyful [46]. *Joy* is also associated with *positive* sentiment [47]. But it may happen that a person is future-oriented, but s/he may always think negative about the future. In that case, although the person is future-oriented, s/he is not joyful. Therefore, bidimensional TP can give us a better insight into human attributes than unidimensional TP.

In the unidimensional measure (similar to [42, 44, 45]), we only consider the different temporal orientations to yield three-factor scale: *Past, Present* and *Future*. However, it is a fact that the tense information present in a sentence does not always incline to actual time information. For example, “*I need your help in the upcoming activity of our group*”. Here, the tense of the verb is *present* but the intended temporal orientation is *future*. To extract the temporal orientation automatically using only the verb tense information is difficult and may require complex hand-crafted rules. It is, therefore, very crucial to design a robust method which would be able to capture the actual temporal orientation from the sentences automatically. It is to be noted that subjunctive sentences are not in the scope of our current study. In our method, we create the training data for classification by aggregating tweets based on Twitter’s hashtag information. Our model then classifies user-level tweets into either of past, present or future categories. Tweets classified in this way are then grouped for each user to get user-level unidimensional TP. The latter measure (i.e. bidimensional TP) takes into account the sentiment view for all the temporal orientations and gives rise of a nine-factor scale: *Past-Positive*, *Past-Negative*, *Past-Neutral*, *Present-Positive*, *Present-Negative*, *Present-Neutral*, *Future-Positive*, *Future-Negative*, and *Future-Neutral*. Finally, we measure the relationship between the users’ TP and their age, education, and six basic emotions.

### Contributions

Our resolution from syntactic tense to semantic time is performed by developing a temporal classifier based on the users’ tweets collected from Twitter. We use an attention-based Bi-directional Long Short Term Memory (Bi-LSTM) network with linguistic feature embedding for the tweet temporal classification. The word-level linguistic features include words having verb Part-of-Speech (Pos) tag and words present in a temporal knowledge-base. We incorporate a hashtag-based minimally supervised method comprising of two-pass filtering to create the *past-*, *present-* and *future*-oriented tweets for the training of the classification network. The performance of the temporal classifier is measured on a manually annotated test set. We then use this classifier to automatically classify a large dataset created by Preoţiuc-Pietro et al. [48] consisting of ≈10 million tweets from 5,191 users of UK population mapped to their age, education and six basic emotions.

The user-level tweets with a particular temporal orientation are further subdivided into either *positive*, *negative* or *neutral* using a pre-trained sentiment classifier to produce bidimensional TP. Finally, we examine how TP (unidimensional and bidimensional) of the users are related to their different attributes. For this study, we consider users’ two demographic attributes (age and education) and six basic emotion categories (joy, sadness, disgust, anger, surprise, and fear) proposed by Ekman [49]. We did not use the extensive emotion categories since the relation between the TP, and the basic emotions have not been explored on a large scale in earlier studies. Please note that our main goal is not to propose a method for emotion detection. We have used the user-level attribute data as created by Preoţiuc-Pietro et al. [48] for our current study.

In summary, our main contributions are as follows:

- We present a method to resolve grammatical tense of users’ tweets into actual time by measuring temporal orientation from tweets.
- We propose a minimally supervised approach for temporal orientation framework that leverages a large quantity of unlabeled data and requires no hand-annotated training corpora. Performance on gold standard data shows that our proposed method outperforms the state-of-art technique;
- We use this method to get the temporal orientation from tweets for a large number of Twitter users and then aggregate over users to obtain users’ unidimensional TP.
- We introduce the valence dimension to assess users’ bidimensional TP.
- We finally explore the relationship between the Twitter users’ TP and their age, education and six basic emotions on a large-scale.

## Method

Our methodology is divided into two parts. The first part deals with the extraction of temporal orientation from users’ tweets and use this information to measure their TP. We then use a pre-trained sentiment classification model [50] to further classify the users’ tweets of each temporal category into *positive, negative* and *neutral*. Second part of the method is to measure the correlations between the users’ TP and their age, education and six basic emotions.

### Extracting temporal orientation from tweets

We build a temporal-orientation classification model based on the attention-based Bi-directional LSTM (Bi-LSTM) network with linguistic feature embedding to classify users’ tweets into three different categories: *past, present* and *future*. We create a dataset following a minimal supervision technique by exploiting the hashtag information for training the classification network. Given the following tweet “*Let me change lanes and turn left legally*”, the temporal orientation classifier should predict it as an instance of *future* orientation.

#### Attention-based Bi-LSTM network with linguistic feature embedding

**Fig 1:**
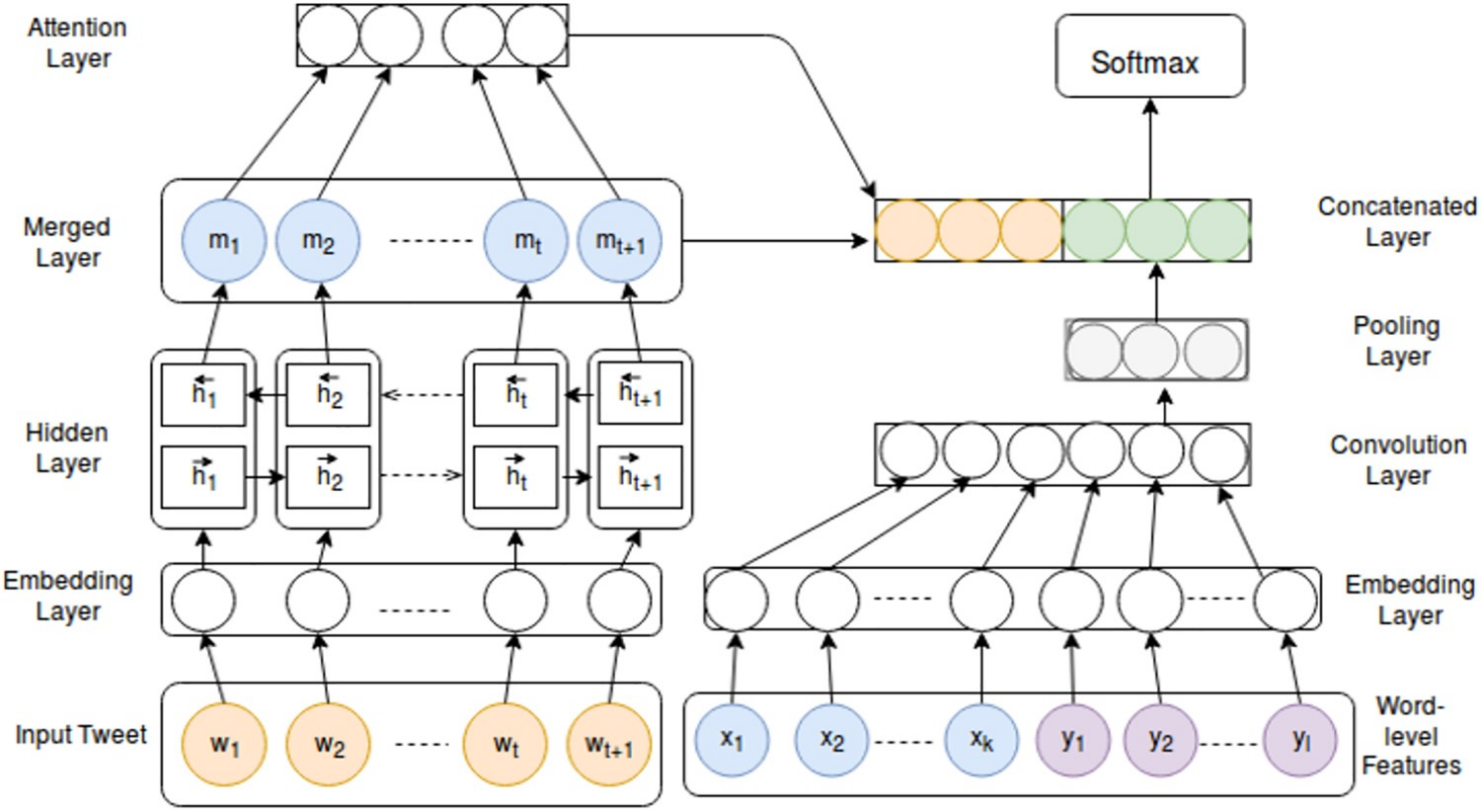
Architecture for temporal orientation classification.

Our proposed temporal classifier is a Bidirectional Long Short Term Memory (Bi-LSTM) [51] network with attention mechanism. We depict the overall architecture in Fig 1. We often fail to capture the temporal orientation of a text using just the tense information or the existing temporal keywords. For example, the tweet “*Today I have a meeting at night*.” is future-oriented. Here, the temporal keyword ‘*Today*’ has a time sense of *present*, whereas the tense of the verb is also *present*. In another example, the tweet “*Working in the same unit today with different staffs was much better*.” has temporal orientation as *past*. Here, the words which have temporal senses (*i.e. working, today, was*) are placed at a distance from each other. Here, the validating temporal information (‘was’ in this case) in the tweet has a long-range dependency with others. Long Short Term Memory (LSTM) [52] has been very useful to capture these kinds of long-range dependencies for the text classification tasks [53]. This motivates us to use the LSTM network.

#### Bidirectional LSTM network

LSTMs are a special kind of recurrent neural network (RNN) [54] capable of learning long-term dependencies in the text by effectively handling the vanishing or exploding gradient problem. At first, a sentence is represented by an embedding layer as a sequence of word vectors *w* = (*w*_1_, *w*_2_, *w*_3_, …, *w*_*N*_), where N is the sequence length. This embedding is then given as input to the Bi-LSTM layer. In the Bi-LSTM, the output of the forward LSTM is represented as $\vec{h_{t}}$ of the sequence from left to right for each word *t*, and the output of the backward LSTM is represented as $\overset{\leftarrow }{h_{t}}$ of the sequence in the reverse direction. Finally, the Bi-LSTM outputs are merged as $m_{t}\;=\left[\vec{h_{t}};\overset{\leftarrow }{h_{t}}\right]$.

#### Attention network

Attention Neural network [55] has been efficient over the LSTM network to find the important segments of a word sequence. The important segments are assigned more weights compared to the others. The merged output representation *m*_*t*_ is given as input to the attention layer and the network produces an attention weight vector *α* and a hidden representation *s*. If *M* is a matrix of the merged output vectors of Bi-LSTM, then *M* can be represented as *M* = (*m*_1_, *m*_2_, *m*_3_, …, *m*_*N*_), where *N* is the sequence length.

We first feed the merged output vector *m*_*t*_ through a non-linear layer to get *p*_*t*_ as a hidden representation of *m*_*t*_ as shown in Eq 1, where *W*_*w*_ and *b*_*w*_ denote to the weights and bias, respectively.

$$\begin{array}{c} p_{t}=tanh\left(W_{w}m_{t}+b_{w}\right) \end{array}$$ (1)

We then measure the importance of the word as the similarity of *p*_*t*_ with a word level context vector *p*_*w*_ and obtain a normalized importance weight *α*_*t*_ passing through a softmax function (Eq 2). The word level context vector *p*_*w*_ is randomly initialized and jointly learned during the training process. It contains contextual information of the merged output layer vectors.

$$\begin{array}{c} \alpha_{t}=\frac{exp({p_{t}}^{T}p_{w})}{\sum_{k}\;exp\left({p_{k}}^{T}p_{w}\right)} \end{array}$$ (2)

After that, we compute the sentence vector *s* as a weighted sum of the word annotations based on the attention weight *α*_*t*_ (Eq 3).

$$\begin{array}{c} s=\sum_{t}\alpha_{t}m_{t} \end{array}$$ (3)

#### Word-level features

We incorporate two word-level linguistic features into the learning process. The first feature corresponds to the verb Part-of-Speech (PoS) information present in a tweet while the second feature is used to capture the temporal keywords present in the tweet. We obtain the PoS information using the CMU tweet-tagger [56]. We capture the temporal keywords in the tweet using a temporal knowledge-base [57]. Finally, the words with verb PoS information and the detected temporal keywords in the tweet are represented in the form of 2D embedding matrices *X* and *Y*, respectively. The embedding matrix *X* ∈ *R*^*d*×*k*^ represents a matrix of embedding dimension *d* (word vector dimension) and feature-length *k* (number of verb PoS tags in a tweet). Each row of the matrix corresponds to a word vector of those words which are detected as verbs. Similarly, the embedding matrix *Y* ∈ *R*^*d*×*l*^ represents a matrix of embedding dimension *d* and feature-length *l* (number of temporal words in a tweet) (c.f. Fig 1). Finally, we maintain an embedding matrix *E* by concatenating two matrices, *X* and *Y* in the lower dimensional space.

We explain our intuition behind using these two word-level features by the following two examples: i). The sentence, “*I have a nice plan for the spring festival*.” has a temporal sense of ‘*future*’ which can be determined by the temporal keyword ‘*plan*’, but not by the verb ‘*have*’. ii). The sentence, “*He lost all the hopes due to his fragile mindset*.” has a temporal sense of ‘*past*’ which can be captured by the verb ‘*lost*’. Here, the temporal keyword ‘*hopes*’ does not help. From these two examples, we can conclude that both the words with verb PoS tags and temporal keywords are important but in different ways.

To get a good feature representation out of these two word-level features, we pass the concatenated embedding matrix *E* through a Convolution Neural Network (CNN). We train CNN with one convolution layer followed by one max pooling layer. In the CNN model, we use 3 filters with window sizes of 5, 6 and 7.

#### Merged output layer

Finally, we concatenate the CNN output *f* with the Bi-LSTM attention output *s*. The concatenated vector (*c* = [*s;f*]) is passed through a non-linear layer for the projection into the space of the targeted temporal classes by the following equation:

$$\begin{array}{c} z_{i}=tanh\left(W_{i}c+b_{i}\right) \end{array}$$ (4)

where *W*_*i*_ and *b*_*i*_ are the weight matrix and bias, respectively. Finally, the class-label is predicted using a softmax function at the output layer (Eq 5).

$$\begin{array}{c} y_{i}=\frac{exp(z_{i})}{\sum_{j}exp\left(z_{j}\right)} \end{array}$$ (5)

#### Model parameters

For our experiments, the loss function we used is *categorical cross-entropy* and the optimizer we used is *Root Mean Square Propagation (rmsprop)*. We repeat the training for 100 *number of epochs* with *batch size* set to 128. We also employ dropout for regularization [58] with a *dropout rate* of 0.2 to prevent over-fitting. All of these parameters are finalized by parameter tuning with the performance obtained on 10-fold cross-validation using the grid search method. The grid search method takes a list of parameters (loss function, optimizer, no of epochs, and batch size) with each parameter having different values (e.g., for the optimizer, values are adam, rmsprop, etc.) and return each parameter with the best possible value based on the accuracy on 10-fold cross-validation. The word vectors of all the words and all the words with verb PoS and the detected temporal keywords in a Tweet are of 200 dimensions. The words are searched in the pre-trained GloVe vector model [59] (trained on 2 billion tweets containing 27 billion tokens). We also validate our model on the validation set which was 10% of the training set. In each epoch, the accuracy is obtained in the validation set. Finally, we fix the model which obtained the highest accuracy on the validation set.

### Measuring user-level TP

Classifying all the tweets of a user by our temporal orientation model, unidimensional TP orientation of that user is defined by the following equation:

$$\begin{array}{c} orientation_{t}\left(user\right)=\frac{|tweets_{t}\left(user\right)|}{|tweets_{all}\left(user\right)|} \end{array}$$ (6)

where *t* ∈ {*past, present*, or *future*}.

The bidimensional TP orientation of a user is defined by the following equation:

$$\begin{array}{c} orientation_{s,t}\left(user\right)=\frac{|tweets_{s/t}\left(user\right)|}{|tweets_{t}\left(user\right)|} \end{array}$$ (7)

where *t* ∈ {*past, present*, or *future*}), and (*s* ∈ {*positive, negative*, or *neutral*} per temporal category. First, we apply the temporal orientation classifier to each user’s tweets. For sentiment classification, we use a pre-trained tweet-level sentiment classification model [50] to classify the *user-level* tweets into positive, negative or neutral. The sentiment classifier was trained on a dataset released by SemEval-2013 shared task. The model was trained with a diverse set of features: word n-grams, character n-grams, PoS, hashtags, sentiment lexicon, emoticons, elongated words and negation. The model achieved the macro-averaged F-score of 69.02%, and ranked first in the SemEval-2013 sentiment analysis task. Finally, for each temporal category, we find the proportion of each sentiment class (i.e positive, negative or neutral) to obtain the bidimensional TP orientation.

### Correlation study

We measure the correlation between the users’ TP with their *age*, *education* and different emotional attributes (*joy*, *sadness*, *disgust*, *surprise*, *anger*, and *fear*) using a linear regression [60] model. The performance is measured using a standard metric, *Pearson’s correlation coefficient r* between the inferred and the target values which measure the linear association between those two values.

## Datasets

For experiments, we categorize the datasets into three kinds: *training, test* and *user-level test set*. Our final training set consists of 45K English tweets, whereas the test set is consisting of 741 manually annotated English tweets. The user-level test set consists of ≈10 million English tweets from 5,191 users from UK population.

### Training set

To collect the data for our hashtag-based minimally supervised method, one key issue is to identify the candidate hashtags. We rely on the trending topics (i.e. hashtags) reported on Hashtags.org website for the candidate identification. The website reports the trending hashtags in three categories: trending up, trending down, and the most popular hashtags. From the trending up and trending down categories, we manually selected those hashtags which signified any temporal (*past, present/ongoing* or *future*) events. To cover more varieties of hashtags in our data collection, we have dropped the most popular hashtags category as it does not change much for many days. We use the selected hashtags as query keywords to search for the tweets on daily basis. On each day, we select the trending hashtags and we then use each selected hashtag as a query keyword to collect the tweets using Twitter’s streaming API. We collected data for two months- September and October, 2017. This collection resulted in over 280K tweets. Few example tweets with the trending topics are depicted in Table 1.

**Table 1:** Example tweets for different temporal orientation categories with trending topics.

Temporal Orientation	Trending Topic	Example Sentence
Past	#CPC17	just heard gazza made a guest appearance outside the tory conference.
Present	#WorldTeachersDay	hats off to all the teachers who work hard to not only educate but protect kids everyday.
Future	#U17WC	2017 fifa u17 world cup starts in 3 days

The collection of the training data are based on the following three hypotheses: (a) if a trending topic is of a future event then people would write mostly futuristic tweets; (b) if a trending topic is about a past incident, then people would write more about the past but they also write about the present effects of that event; (c) the tweets of the trending present event are the most critical to handle as besides writing about the present incidents, people sometimes refer to the past incidents and also give opinion about the future effects.

To deal with the pitfalls described in the hypotheses, we filter the collected tweets using a two-pass filtering method. The method is based on two assumptions, *viz*. (a) every meaningful sentence should contain a verb; (b) most of the past-oriented tweets contain a verb of the past tense. After two-pass filtering, our collection resulted in over 72K weakly labeled tweets: 24K *Past*, 29K *Present*, and 19K *Future*. We call these tweets as weakly labelled tweets since the labels are not manually annotated, instead generated based on the manually selected hashtags which are representatives of past, present, and future temporal categories.

The first assumption is well-established in the literature, whereas the second assumption is based on our observation on the tweets and validation against a tense-based classifier. In the first pass of the filtering method, we remove the tweets which do not contain any verb. We obtain this PoS tag using the CMU tweet-tagger [56]. In the second pass of the filtering method, we remove the tweets having tense as *past* from the tweets of *present* and the *future* events.

The CMU tweet-tagger does not provide verbs in different sub-categories. For this reason, we also obtain the PoS tag information from the Stanford PoS-tagger [61]. This provides the subcategories of verbs (i.e. VB-Verb base form, VBD-Verb past tense, VBG-Verb gerund or present participle, VBN-Verb past participle, VBP-Verb non-3rd person singular present, and VBZ-Verb 3rd person singular present). We observed that although the Stanford PoS-tagger assigned the required verb subcategories, it also introduced false positives, i.e. incorrectly tagged some non-verbs as verbs. This is the reason why we considered only those verbs (for sub-categorization) which were identified (as verbs) by the CMU tweet-tagger.

### Test set

We evaluate our temporal-orientation classifier on a manually created test set. Three annotators (post-graduate level students and native English speaker) were asked to tag the 800 randomly selected English tweets in one of the four available classes, namely *past*, *present*, *future* and *other*. The annotation guidelines were as follows:

1. Tag a tweet as *past* if it talks about an event which has started as well as ended or the underlying temporal connotation of the tweet refers to the *past* time.
2. Tag a tweet as *present* if it talks about an event which has started but not ended yet or the tweet has a *present* temporal connotation.
3. Tag a tweet as *future* if it talks about an event which is yet to happen.
4. Tag a tweet as *other* in case they found it difficult to get the exact temporal tag for the tweets.

We measure the multi-rater kappa agreement [62] among the annotators. Similar to [44], we found to have a substantial agreement (kappa value of 0.82) between the annotators for the task. Finally, we select the temporal class of a tweet based on the majority voting among the annotators. The class distribution are as follows: 375 *Past*, 164 *Present*, 202 *Future*, and 59 *Other*. For our final evaluation, we remove the *Other* cases from the test set and thus we use 741 tweets as the test set.

### User-level test set

The dataset is developed by Preoţiuc-Pietro et al. [48]. It consists of ≈10 million tweets from 5,191 users mapped to their user-level age, education and six basic emotion features: *joy, sadness, disgust, surprise, anger* and *fear*. The authors used various models such as Logistic Regression, Support Vector Regression and Gaussian Process for Regression to train with the lexical features extracted from the tweets to predict the emotion of each tweet. In addition to lexical features a set of stylistic features including emoticons, elongated words, capitalization, repeated punctuation, number of hashtags and the clause level negation were also used to build the predictive model. Finally, all the emotions per user were aggregated and the proportion of every emotion per user was calculated. Their emotion prediction model achieved an overall accuracy of 78% on a benchmark dataset.

## Results

In this section, we present the results of temporal orientation classification and present the details of correlation studies.

### Results of temporal orientation classification

Our temporal orientation classifier measures the orientation of each tweet in either of the *past*, *present* or *future*. The classifier was trained on the training set and evaluated on the manually annotated test set. We varied the training data size starting from 3K (equally distributed among three temporal classes) to 72K (overall collection after two-pass filtering) tweets and measured the accuracy over manually annotated 741 test samples. We obtain the highest accuracy of 78.7% with 45K training instances, equally distributed among the *past*, *present* and *future*. We define the baseline model based on the recent state-of-the-art system [45] where a temporal keyword-based weakly-supervised approach was followed to create the training set. The features were extracted using CNN, and these were used for training an SVM classifier. We present the comparative results of temporal orientation classification task in Table 2.

**Table 2:** Accuracy for *past, present, future* classifications using different methods measured over test data. Results are broken down by precision (p), recall (r), and F1-measure.

Method	Baseline	Proposed Method		
		all features	w/o keywords	w/o verbs
**Accuracy**	67.1	**78.7**	74.9	75.3
Past (p, r, f1)	(82.79, 68.00, 74.67)	(82.82, 92.53, 87.41)	(84.93, 82.66, 83.78)	(83.95, 89.33, 86.56)
Present (p, r, f1)	(42.41, 75.00, 54.18)	(82.00, 50.00, 62.12)	(61.90, 55.48, 58.52)	(66.67, 50.00, 57.14)
Future (p, r, f1)	(81.11, 57.42, 67.24)	(69.37, 76.24, 72.64)	(67.25, 76,24, 71.46)	(64.38, 69.80, 66.98)

We perform feature ablation study to understand the significance of each feature. The results are reported in Table 2. Here, we find that we get the best result when we use both the features together. Results in Table 2 show that our proposed method is the most effective in correctly classifying the *past* class followed by the *future* and *present*. We observe low recall for the *present* class as many tweets which belong to *present* are misclassified into either of *past* or *future* classes. One reason may be the fact that the words in the tweet which represent present tense are not in the right form (Its, theres). The *present* tweets are misclassified into the *past* in those cases where mainly the existence of the tense of a verb is *past*, but actually the tweet has *present* orientation. The tweets with *future* orientation are mostly misclassified into the *past* orientation. These kinds of misclassifications are either due to the presence of past tense or the tweet is a compound sentence which has an independent clause referring to the past orientation.

Our further analysis on tense to time disambiguation shows that in only 21.6% of the manually annotated test tweets time information is resolved using only the tense information. For the remaining tweets, tense information alone is not helpful. In those remaining test tweets, our temporal orientation classifier is able to correctly disambiguate time information for 77.3% tweets. This shows that our method can resolve many instances where only tense information cannot help. Our method miss-classifies the tweets where the tweet, itself, a bit confusing and complex. For example, the tweet “*Laundry day Besides the Dr Horrible song other phenomena that I enjoy involves just perusing the Juke Box-the songs just play in my head*.” is present-oriented but our method classifies it as past.

### Results of user-level TP

**Fig 2:**
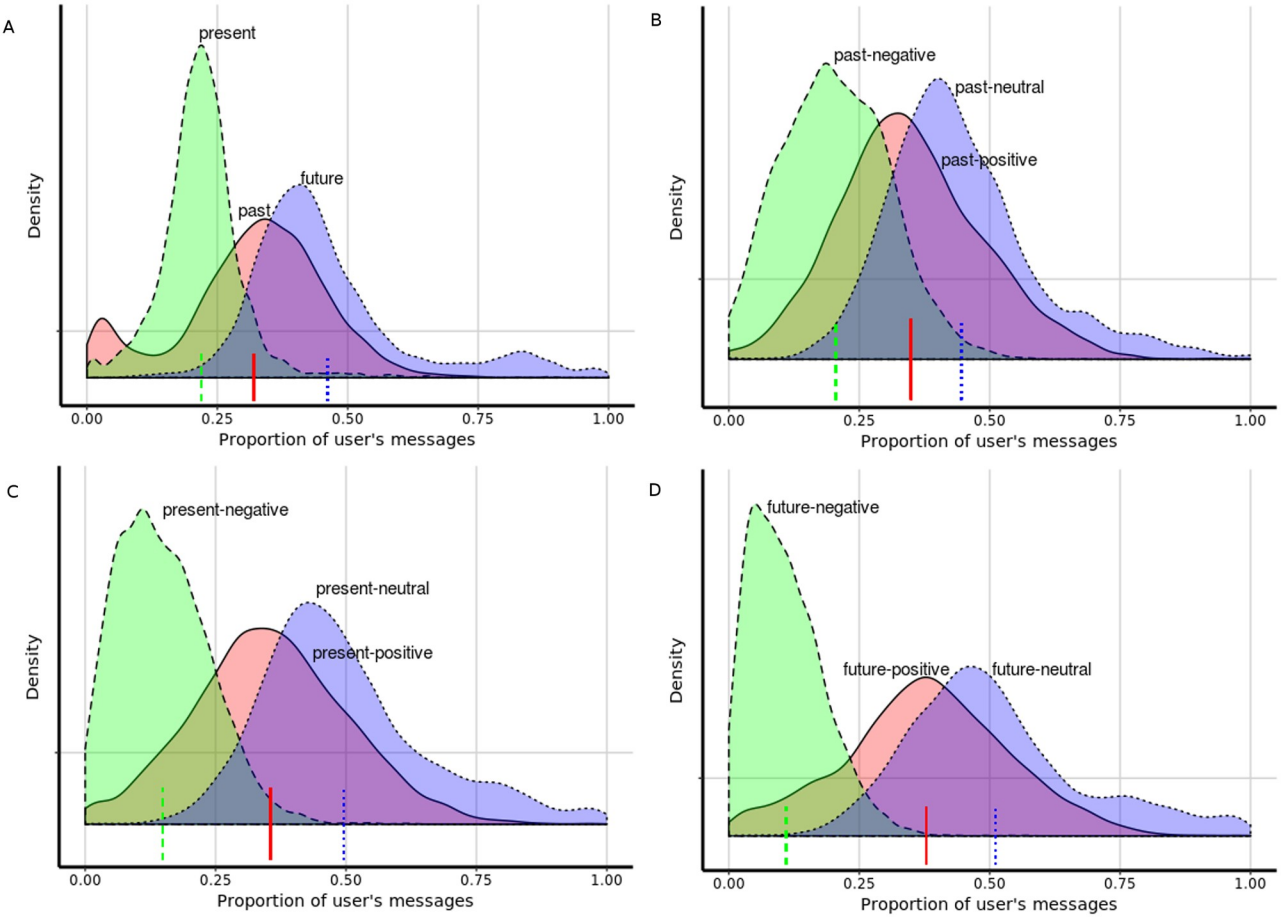
Kernel density estimate of user-level orientation. (A) Kernel density estimate of user-level orientation of past, present and future; (B) Kernel density estimate of user-level orientation of past-negative, past-positive and past-neutral; (C) Kernel density estimate of user-level orientation of present-positive, present-negative and present-neutral; (D) Kernel density estimate of user-level orientation of future-positive, future-negative and future-neutral.

Afterwards, all the results and analysis are on the user-level test set. Fig 2 illustrates the user-level distribution of the tweets’ classes.

The mean proportions of users’ tweets are shown in Table 3. Within users, the mean proportions of *past*, *present*, and *future* tweets were 0.32, 0.22 and 0.46, respectively. Among most users, the majority of messages are classified as *future*, while present-oriented messages are the least frequent. Within users’ *past*, *present* and *future* orientation, neutral messages were the most frequent while negative messages were the least frequent.

**Table 3:** The mean proportions of users’ tweets into different TP categories.

past			present			future		
0.32			0.22			0.46		
**positive**	**negative**	**neutral**	**positive**	**negative**	**neutral**	**positive**	**negative**	**neutral**
0.35	0.21	0.44	0.36	0.15	0.49	0.38	0.11	0.51

### Correlation results and discussion

We measure the predictive power of TP by performing regression on different users’ attributes. We analyze the correlation between the users’ TP and their age, education and six basic emotions. In this section, all the discussions and analyses are based on the correlation results over the user-level test set. All the correlation coefficient values are found to be statistically significant by Fisher’s R-to-Z transformation (Bonferroni corrected) with *p* <.001.

**Fig 3:**
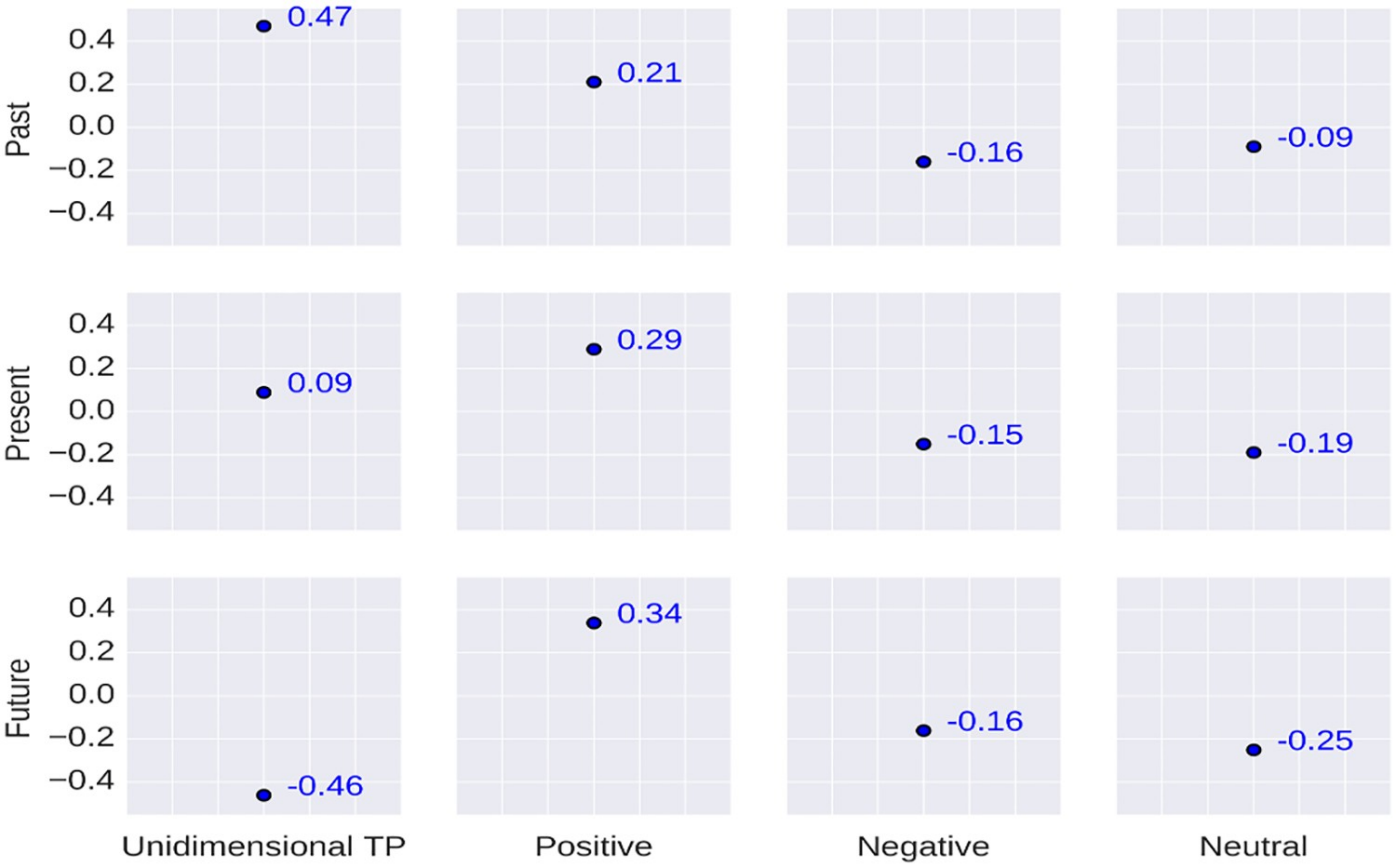
Correlation between the users’ TP and their age. The Y-axis values represent the correlation coefficients. The first column represents the correlation coefficient between users’ unidimensional TP and their Age. Rest entries are for bidimensional TP vs Age (Eg. First row, second column entry represents the correlation coefficient value between past-positive and Age).

The correlation between the users’ age and their TP is shown in Fig 3. Evaluation results show that the users’ *past* orientation is positively correlated with age (*r* = 0.47). It signifies that people become past oriented when they age. This is in-line with a recent literature [44]. It is also to be noted that the users’ age is negatively correlated with *future* orientation (*r* = -0.46) which suggests that the users think less about the future when they age. Considering the valence dimension we obtain a correlation coefficient of *r* = 0.34 between the users’ *future-positive* orientation and their age which implies that users become more positive about the future when they age.

**Fig 4:**
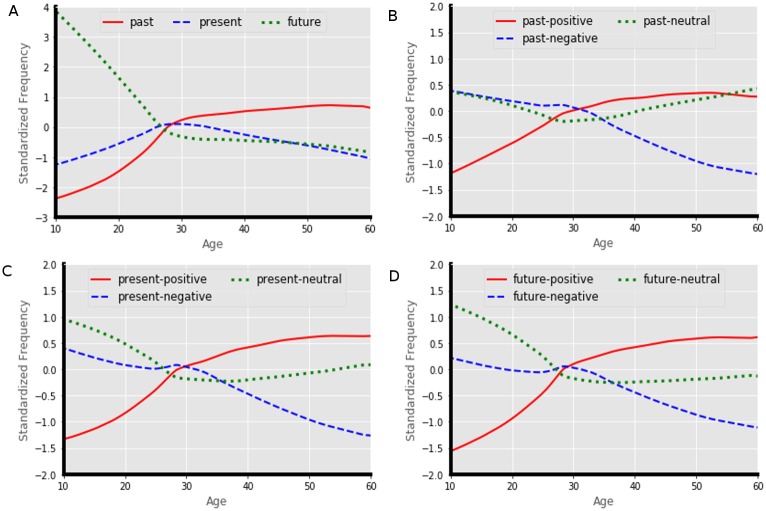
The users’ standardized frequency of TP over their age. Smoothing performed using loess [63] smoothing estimates. (A) The users’ standardized frequency of past, present and future orientation over their age; (B) The users’ standardized frequency of past-negative, past-positive and past-neutral over their age; (C) The users’ standardized frequency of present-positive, present-negative and present-neutral over their age; (D) The users’ standardized frequency of future-positive, future-negative and future-neutral over their age.

The users’ standardized frequency of TP over their age is shown in Fig 4. We observe in Fig 4A that the trend of users’ temporal orientation changes after the age of 27 to 28. Users’ *past* orientation increases sharply until this period and then increases steadily. Users’ *present* orientation increases steadily till this period and then also decreases steadily. The *future* orientation of the user decreases sharply till this period and then becomes almost stable. Fig 4B shows that the users’ orientation of *past-positive* changes steadily with age. The *past-negative* orientation decreases with the increase of age while the *past-neutral* orientation decreases until age 30 and then increases steadily. We observe the same kind of patterns in Fig 4C and 4D where a change of trend is also seen near the age of 30.

**Fig 5:**
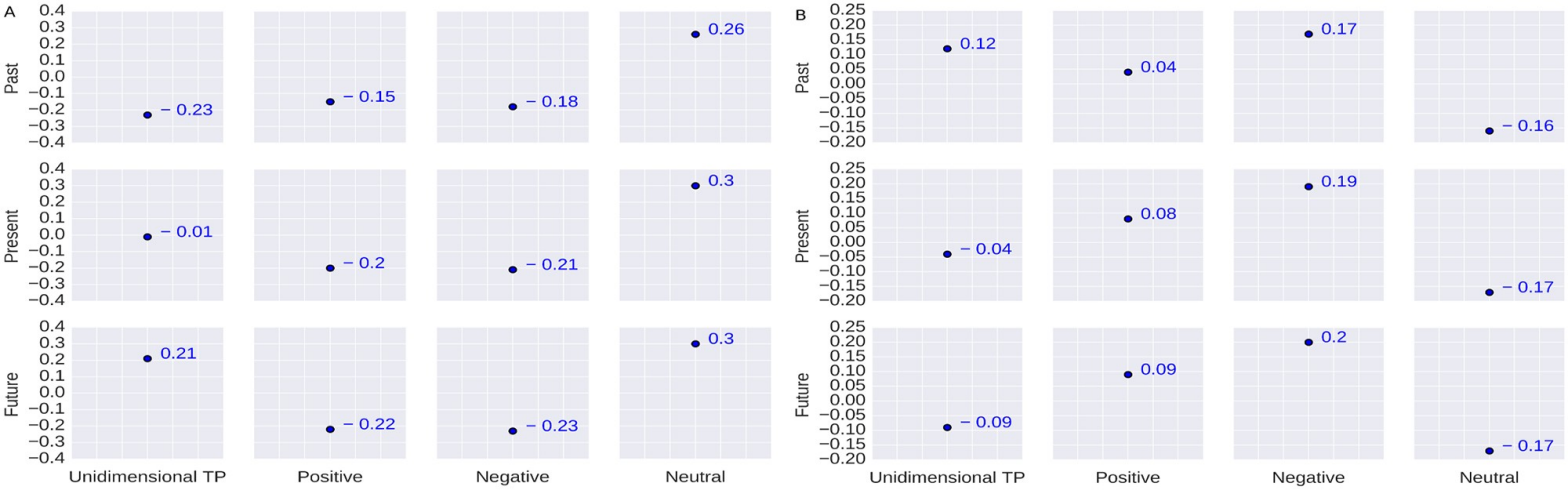
Results of correlation coefficient. (A) Correlation between the users’ TP and Education:graduate_degree; (B) Correlation between the users’ TP and Education:high_school.

We measure the correlation between the users’ TP and their education in two sub-categories: education:graduate_degree, and education: high_school. The correlation results are shown in Fig 5.

Literature suggests that students’ temporal orientation can be useful for analyzing their academic engagement [64, 65]. In the psychological literature, education has been associated with the *future* orientation [64]. Our empirical results depicted in Fig 5A show that users with education as graduate_degree are future oriented (*r* = 0.21). The addition of valence dimension suggests that users with education graduate_degree are relatively more neutral about their *future* orientation (*r* = 0.30). Users with education high_school are seemed to be past oriented (*r* = 0.12) (Fig 5B). However, when we add the valence dimension we observe that these users are relatively more *future-negative* oriented (*r* = 0.20).

**Fig 6:**
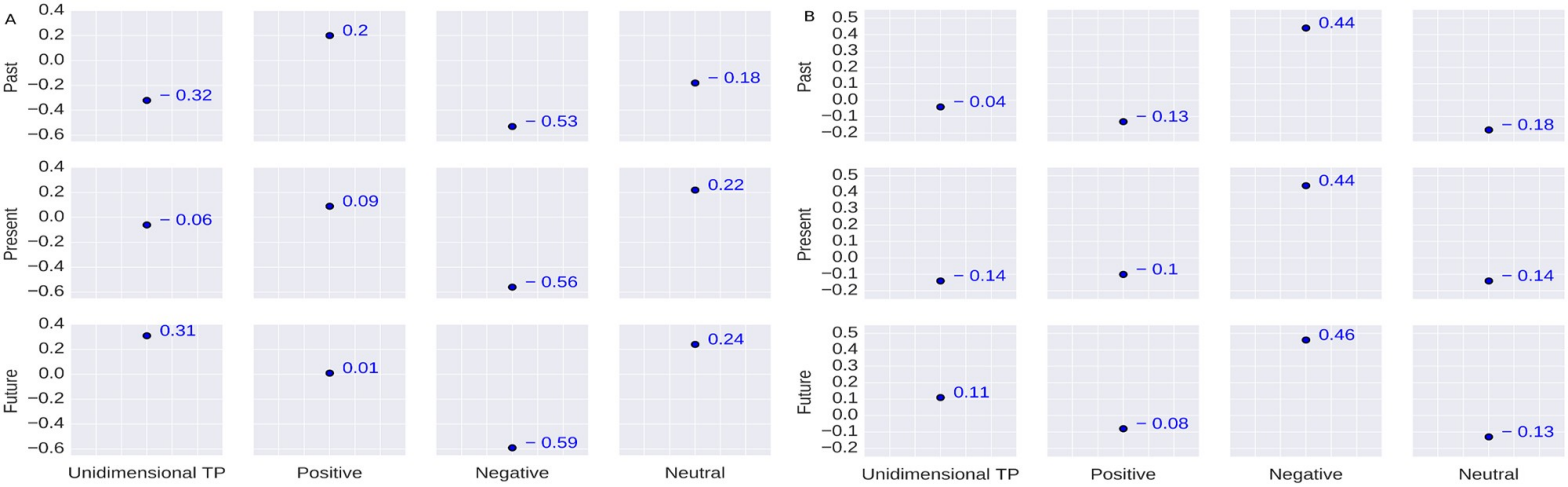
Correlation coefficient results. (A) Correlation between the users’ TP and Joy; (B) Correlation between the users’ TP and Sadness.

The first emotional attribute we consider is *joy*. In the psychological literature, we found that *joy* was related to *future* TP [46]. It was also associated with *positive* sentiment [47]. The result in Fig 6A shows that there is a positive correlation between *future* TP and *joy* (*r* = 0.31). We also find that *joy* has a negative correlation with the *present* and *past* orientation. It indicates that the *future*-oriented people are more joyful which is in-line with the literature. In case of bidimensional TP, we find that joyful people are more *neutral* about the *future* which is not the case found in the literature (joy vs. positive sentiment). One possible reason may be due to the miss-classifications of many *positive* classes into *neutral* by the sentiment classification model.

The second emotional attribute *sadness* was associated with *past* and *present* orientation [66, 67] in the psychological literature. It was also associated with *negative* sentiment [68]. The results in Fig 6B show that *sadness* has a higher correlation with *future-negative* followed by *past-negative* and *present-negative* which indicates that the *sad* people are *negative* minded. Moreover, they are relatively more negative about the *future* followed by the *past* and the *present*. In this case, considering only the unidimensional TP is somehow misleading as we see that *sadness* is positively correlated to the future (*r* = 0.11) but negatively correlated to the *past* and *present* orientation.

**Fig 7:**
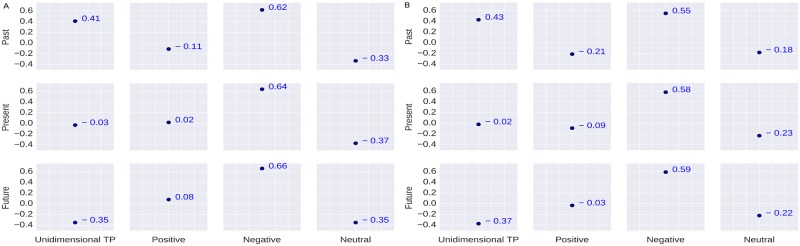
Correlation coefficient results. (A) Correlation between the users’ TP and Disgust; (B) Correlation between the users’ TP and Anger.

Our experimental results in Fig 7A show a positive correlation for *disgust* with the *past* (*r* = 0.41) and negative correlations with the *present* and *future* which indicates that *past* oriented people are more *disgusted*. When we consider bidimensional TP, we find relatively higher correlation between *future-negative* and *disgust* (*r* = 0.66) which indicates that people with *disgust* emotion possess more *negative* view of the *future*. In literature, *disgust* was related to *negative* sentiment [68] while the relationship between disgust and the temporal orientation was not clear.

In the psychological literature, *anger* was associated with the *present* orientation [38, 67, 69] and *negative* sentiment [67, 68, 70]. In some of the prior studies, it has been shown that *anger* and *joy* are similar in case of emotional arousal but different in valence (negative and positive, respectively) [71]. The study presented in [67] has shown that impact of memories of the negative and catastrophic past events can evoke negative emotion like *anger*. This suggests that there is also a relationship between *anger* and *past* orientation. Our experimental results in Fig 7B show that *anger* has a positive correlation (*r* = 0.43) with the *past* orientation and negative correlation with the *present* and *future* orientation. In case of bidimensional TP, we find that the angry people express relatively more *negative* sentiment towards the *future* (*r* = 0.59) than the *past* and the *present*.

**Fig 8:**
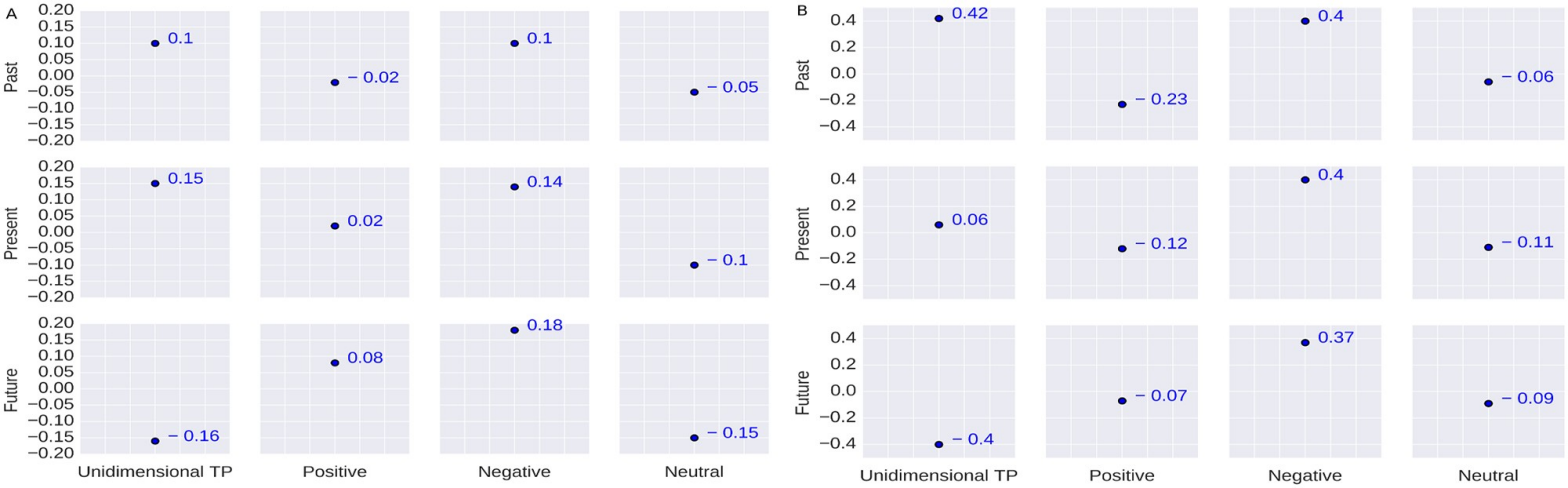
Correlation coefficient results. (A) Correlation between the users’ TP and Surprise; (B) Correlation between the users’ TP and Fear.

The relationship between the emotional attribute *surprise* and the TP is not extensively studied in the literature. We find a positive correlation value (*r* = 0.15) between *present* orientation and *surprise* as shown in Fig 8A. It signifies that the present TP is more related to surprise. Some literature say that *surprise* is related to both the *positive* and *negative* sentiment based on the pleasantness and unpleasantness of the incident related to surprise [72]. We find that people while surprised think relatively more *negative* about the *future* (*r* = 0.18) than the *present* and the *past*.

Fear is an emotional arousal of something which does not exist at present. In the psychological literature, *fear* is associated with negative sentiment [68, 70] and is said to occur in response to a pending mismatch [71] or generated by an anticipated state [67, 73, 74]. The results in Fig 8B show that *fear* is correlated with the past TP (*r* = 0.42). However, when we consider bidimensional TP, we find that *fear* has relatively higher correlation with the *past-negative* followed by the *present-negative* and *future-negative*.

Although some of the findings are linked to the literature, but those research were done at the psychological or theoretical level for a small number of users. In contrast, our study conducts a large scale empirical evaluation, and finds the correlation for a large number of users based on their social media tweets.

## Limitations and future scope

Here, we acknowledge the possible limitations of this study. Firstly, (i). We have not considered atemporal (where the sentence has no time sense involved) class as a separate class from the present. For example, the sentence “*The color of this table is red*.” has no time sense but some may argue and consider this as an instance of the present. (ii). There are some sentences like “*You have this tendency to lie often*.” may seem to have a present time sense but it may be categorized in a separate class from the present. (iii). There are examples like “*He will think that she is pregnant*.” has present or future orientation depending upon the context in which the sentence is used. As our method gives only a single tag to each sentence, it will be classified in either of present or future class depending upon the pattern learned while training. Still, our tense to time resolution method has been able to resolve 78.7% of the test instances.

Secondly, our method may suffer in terms of a user’s true identity (fake account). The demographic correlations also may subject to differ depending upon the demography itself. We leave it to the scope of future works. In the future, an alternative approach for classifying the tweets can be explored for a more accurate measure of TP. In future, we shall focus on improving the performance of temporal orientation classifier as well as sentiment classifier for this purpose.

## Conclusion

In this article, we have presented a very first large-scale study to assess unidimensional and bidimensional TP from the language of social media tweets. We used tweets as these are easy to access, and provide potential information to study human cognition and tendencies. For this, extracting actual time information from tweets by tense to time disambiguation become the foremost task as for many tweets grammatical tense and syntactic time is not the same. Our temporal orientation model achieves an accuracy of 78.7% on a manually annotated test set. Moreover, our approach shows how computational techniques can be effectively used to study human cognition like TP. Whereas the previous computational large-scale studies on TP concentrated on the unidimensional measure, we focused on both the unidimensional and the bidimensional TP. We finally investigated the relationship between Twitter users’ TP and their age, education and six basic emotions. This study opens up many aspects of social and psychological science which were not possible previously on a large scale. For example, how people’s TP changes over their lifetime can be an interesting way to address the effects of TP on health and psychology.
